# Relevant Membrane Transport Proteins as Possible Gatekeepers for Effective Pharmacological Ascorbate Treatment in Cancer

**DOI:** 10.3390/antiox12040916

**Published:** 2023-04-12

**Authors:** Christian Leischner, Luigi Marongiu, Alban Piotrowsky, Heike Niessner, Sascha Venturelli, Markus Burkard, Olga Renner

**Affiliations:** 1 Institute of Nutritional Sciences, Department of Nutritional Biochemistry, University of Hohenheim, Garbenstraße 30, 70599 Stuttgart, Germany; 2 Department of Internal Medicine VIII, University Hospital Tuebingen, Otfried-Mueller-Straße 10, 72076 Tuebingen, Germany; 3 Department of Dermatology, Division of Dermatooncology, University of Tuebingen, Liebermeisterstraße 25, 72076 Tuebingen, Germany; 4 Cluster of Excellence iFIT (EXC 2180) “Image Guided and Functionally Instructed Tumor Therapies”, 72076 Tuebingen, Germany; 5 Institute of Physiology, Department of Vegetative and Clinical Physiology, University of Tuebingen, Wilhelmstraße 56, 72074 Tuebingen, Germany

**Keywords:** ascorbate, cancer, ferroptosis, iron, labile iron pool, transporter, tumor, vitamin C

## Abstract

Despite the increasing number of newly diagnosed malignancies worldwide, therapeutic options for some tumor diseases are unfortunately still limited. Interestingly, preclinical but also some clinical data suggest that the administration of pharmacological ascorbate seems to respond well, especially in some aggressively growing tumor entities. The membrane transport and channel proteins are highly relevant for the use of pharmacological ascorbate in cancer therapy and are involved in the transfer of active substances such as ascorbate, hydrogen peroxide, and iron that predominantly must enter malignant cells to induce antiproliferative effects and especially ferroptosis. In this review, the relevant conveying proteins from cellular surfaces are presented as an integral part of the efficacy of pharmacological ascorbate, considering the already known genetic and functional features in tumor tissues. Accordingly, candidates for diagnostic markers and therapeutic targets are mentioned.

## 1. Introduction

For 2020, the cancer incidence, estimated for 36 cancer types in 185 countries, was about 19.3 million with a total of nearly 10.0 million deaths [1,2]. Worldwide, 28.4 million new cancer cases are expected for the year 2040, a 47% increase from 2020 [2]. A detailed cancer diagnosis is essential for appropriate and effective treatment including cancer phenotype, tumor stage, and the personal circumstances of patients. Some of the most common cancer types have high cure probabilities when detected early and treated accordingly. Unfortunately, a significant variation in treatment availability exists between countries of different income levels. Comprehensive treatment is reportedly available in more than 90% of high-income countries but in less than 15% of low-income countries [3]. Remarkably, small cell lung cancer (SCLC), pancreatic ductal adenocarcinoma (PDAC), advanced ovarian cancer (AOC), triple-negative breast cancer (TNBC), and glioblastoma (GBM) are very aggressive solid tumors displaying highly invasive phenotypes and treatment resistance [4]. For these tumor entities, there is currently an urgent need for novel treatment approaches. Vitamin C (ascorbic acid, ascorbate) is not only an essential human micronutrient [5] with a recommended daily intake of 110 mg [6] but also a bioactive substance acting as a prodrug, e.g., for the formation of hydrogen peroxide (H_2_O_2_), especially in pharmacological concentrations, intravenously administrated in the range of grams per kilogram bodyweight. Intravenous ascorbate is widely used by complementary and alternate medicine practitioners, commonly to treat infectious diseases, cancer, and fatigue [7]. After excluding potential contraindications, such as glucose-6-phosphate dehydrogenase deficiency, impaired renal function, and kidney stones, intravenous high-dose vitamin C treatment is an option [8,9]. Sealed vitamin C solutions are stable at room temperature [10] and moreover, vitamin C is very cost-effective and globally available. These criteria make the exploration of parenteral high-dose vitamin C a promising approach for cancer therapy [10,11] which is currently being evaluated in clinical trials. In the early 1970s, it was successfully demonstrated that intravenous administration of high-dose ascorbate contributes to a significant prolongation of survival in end-stage cancer patients [12,13,14]. Interestingly, the peak plasma concentration found after ascorbate ingestion was 220 μM [15]. Liposomal formulations may result in slightly higher peak plasma ascorbate concentrations after oral application by increasing the plasma half-life and by enhancing bioavailability [16]. Nevertheless, antitumoral activity seems to be mainly obtained in the millimolar range which can only be achieved parenterally [17,18,19]. Accordingly, pharmacologic vitamin C as mono- or combination therapy is described as requiring i.v. administration of up to 1.5 g vitamin C per kilogram body weight, yielding plasma concentrations of ≥20 mM [20]. It is suggested that this infusion should be performed at least two times a week for a minimum of eight weeks, according to the evaluation of 71 preclinical and 57 partly ongoing early clinical trials [21]. However, confirmatory placebo-controlled double-blind studies on the efficacy and tolerability of ascorbate use in larger patient cohorts are currently still lacking for definitive proof and implementation of pharmacological ascorbate’s use in tumor therapy. Nevertheless, ascorbate therapy was shown not only to be well-tolerated but also to relieve pain and to improve quality of life in the context of palliative care [7,20,21,22]. Combined treatments, comprising standard treatment protocols (chemotherapy, radiotherapy, targeted therapy, and others) and high-dose vitamin C have mostly been shown to improve therapeutic efficacy, disease control, and objective response rates in some early clinical studies of small study cohorts [7,20,21,22]. The anti-cancer mechanisms by which vitamin C acts on malignant cells include immune modulatory effects, epigenome regulation, collagen synthesis, inhibition of epithelial-mesenchymal transition (EMT) and invasion, and pro-oxidant activity [21]. The most frequently described mechanism is a selective cytotoxic effect on cancer cells (pro-oxidative), which increases the redox imbalance and causes oxidative stress, DNA damage, and an arrest of anti-oxidative enzymes, underlining the various antitumoral effects of vitamin C in relation to the respective treatment [23,24,25,26,27,28,29,30]. However, knowing the circumstances under which vitamin C and its contributors are able to enter tumor cells is fundamental. This review provides an overview of the major membrane conveying proteins implicated in the anticancer action of high-dose ascorbate and co-players that must enter the cell, particularly focusing on the pro-oxidant facet and highlighting the relevant transport systems required for this treatment strategy. In this context, the current knowledge gaps are addressed, and an outlook on future perspectives of high-dose vitamin C in the context of therapeutic approaches is provided.

## 2. Generation of Reactive Oxygen Species and Intracellular Toxicity

Vitamin C is a water-soluble ketolactone with two ionizable hydroxyl groups [31]. In the absence of catalytic metals, the spontaneous oxidation of ascorbate proved to be quite slow in various buffer solutions at pH 7.0 [32]. The dominant species for vitamin C at pH 7.0 are mainly ascorbate (AscH^−^ (99.9%)), its protonated form AscH_2_ (0.1%), and the dianion Asc^2−^ (0.005%) [31,33]. The predominant mechanism underlying the anticancer activity of parenteral pharmacological vitamin C is based on its ability to act as a prodrug [34,35] due to preferential steady-state formation of the ascorbate free radical (AFR; Asc•^−^) and H_2_O_2_ in the extracellular space but minimal formation in the blood, requiring a threshold Asc•^−^ concentration of at least ≈ 100 nM [15,16,35]. In the blood, Asc•^−^ formation is inhibited by red blood cell membrane-reducing proteins [36] and H_2_O_2_ is immediately degraded by plasma catalase and red blood cell glutathione peroxidase [34,37,38]. Asc•^−^ formation exponentially correlates to an increasing ascorbate concentration in the extracellular fluid. The lost electron reduces a protein-centered iron atom and donates an electron to oxygen, forming superoxide (O_2_•^−^) with subsequent dismutation to H_2_O_2_ extracellularly [35,39]. Plasma membrane-associated nicotinamide adenine dinucleotide phosphate (NADPH) oxidases (NOXs) can also contribute to the formation of O_2_•^−^ that dismutates to H_2_O_2_ [40], which can freely diffuse across the plasma membrane or enter the cytosol through peroxiporins.

Generally, members of the NOX family and a considerable number of mitochondrial respiratory chain oxidases are the main generators of H_2_O_2_ [41,42,43,44,45,46]. Additionally, other cellular organelles also contribute to H_2_O_2_ production, e.g., the endoplasmic reticulum and peroxisomes [47,48,49,50]. At low intracellular concentrations, H_2_O_2_ acts as a signaling agent that, e.g., promotes proliferation and survival [45,51,52]. However, H_2_O_2_ concentrations produced by pharmacological ascorbate injection are higher than survival-promoting H_2_O_2_ concentrations, leading to cell death instead [35]. Among the various oxygen metabolites, H_2_O_2_ is considered to be the most suitable for redox signal transduction and also modulates the activity of transcription factors [53]. Further, mitochondrial H_2_O_2_ metabolism may also affect calcium signaling [54]. 

Originally, ascorbate was thought to have prodrug activity, as also suggested by Chen et al. [35], due to selective H_2_O_2_ formation in the extracellular space, but not in the blood, triggered by external pharmacological ascorbate concentrations [34]. In addition, there is evidence that when cells are exposed to an external source of H_2_O_2_, the rapid degradation of H_2_O_2_ inside the cell provides the driving force for the formation of the gradient across the plasma and other subcellular membranes [55]. The steady-state concentration of H_2_O_2_ in intact cells was calculated to be about 1–10 nM [46]. Supraphysiological concentrations of H_2_O_2_ (>100 nM), e.g., induced by high-dose ascorbate, lead to intracellular accumulation of H_2_O_2_, destruction of biomolecules, disrupted redox signaling, cell growth arrest, and cell death [34,46]. Therefore, H_2_O_2_ uptake and distribution in cells and tissues are subject to gradient kinetics (gradients between extracellular and intercellular as well as between subcellular cellular compartments [46]). 

The initiated formation of extracellular H_2_O_2_ promotes its accumulation in tumor tissue [56]. Accumulation of cellular H_2_O_2_ mediates increased toxicity in sensitive cells and displays oxidatively modified proteins in mitochondrial fractions correlating with a decline in the intracellular ATP level [57] via multiple pathways [35]. For example, H_2_O_2_ can cause DNA single-strand breaks that are usually repaired by polyADP-ribose polymerase (PARP), but increased PARP activity can consume intracellular nicotinamide adenine dinucleotide (NAD^+^), leading to ATP depletion [58,59]. In addition, cancer cells that rely on anaerobic metabolism for ATP generation (Warburg effect) are deprived of glucose [60]. This is because the degradation of H_2_O_2_ in cells is mediated in part by glutathione (GSH) peroxidase. However, GSH peroxidase has a high requirement for GSH, which is oxidized to GSH disulfide (GSSG) during enzyme activity. GSSG is regenerated with reducing equivalents from NADPH to GSH, which in turn is regenerated from glucose via the pentose shunt. Glucose, which is used to reduce NADP^+^ to NADPH, is therefore inaccessible in most malignant cells for ATP formation [58]. Overall, these findings strongly support the hypothesis that mitochondrial O_2_•^−^ and H_2_O_2_ significantly contribute to the loss of glucose to the pentose shunt, leading to a decrease in ATP, enhancing cytotoxicity and metabolic oxidative stress in human cancer cells [61,62,63]. However, the subsequent NAD^+^ depletion and energetic crisis are dependent on the specific tumor genotype [22,64]. Moreover, mitochondria in some cancer cells exhibit increased sensitivity to hydrogen peroxide and may be less efficient at ATP generation than normal cells [62,65,66]. Such increased sensitivity of mitochondria to H_2_O_2_, with or without inefficient baseline ATP production, may also lead to decreased ATP production. These pathways of ATP depletion induced by H_2_O_2_ could be independent or more than one could be responsible for cell death in sensitive cells [62,65]. Because primary ATP generation occurs in normal cells via aerobic metabolism, and their mitochondria may not be as sensitive to H_2_O_2_ as those of some cancer cells, these cells would not be affected by pharmacological ascorbate-induced H_2_O_2_. In summary, cancer cells that largely use oxidative phosphorylation to generate ATP may be more sensitive to pharmacological ascorbate compared with cancer cells that are predominantly glycolysis-dependent [67]. Pharmacological ascorbate generates extracellular H_2_O_2_ as an essential initiator of subsequent pro-oxidative damage. However, there is evidence that catalase, by disproportionating H_2_O_2_, blocks the effects of pharmacological vitamin C [22,46,68,69]. Notably, increased levels of different NOXs at the tumor site constitute reliable prognostic markers in human gastric cancer [70,71]. NOX-derived reactive oxygen species (ROS) were shown to be a contributor to tumor development, proliferation, invasion, metastasis, and tumor-mediated angiogenesis [72]. As most tumors have decreased ability to metabolize H_2_O_2,_ due to inefficiency or absence of H_2_O_2_ metabolizing enzymes, malignant cells are susceptible to pharmacologic ascorbate [73]. Moreover, H_2_O_2_-induced spatiotemporal changes in intracellular labile iron trigger the destabilization of lysosomal compartments, promoting a concomitant early response of proteins of iron homeostasis [74]. The intracellular labile iron pool (LIP) [75] is an important determinant of cellular response to oxidative stress [74,76,77]. Schoenfeld et al. showed that O_2_•^−^ and H_2_O_2_, derived from increased mitochondrial metabolism, can increase pools of free, unbound cytosolic iron [22]. This pharmacological ascorbate-induced LIP increase contributes significantly to the cancer cell-selective toxicity of pharmacological ascorbate. The increased LIP in cancer cells in turn contributes to increased oxidation of ascorbate in the cell, generating further H_2_O_2_. This exacerbates the differences regarding labile iron in cancer cells compared to normal cells. Moreover, this may be due to the H_2_O_2_-mediated destruction of iron–sulfur cluster-containing proteins [78]. In addition, the increased H_2_O_2_ concentrations in the presence of elevated LIP may contribute to enhanced Fenton chemistry that generates hydroxyl radicals and causes oxidative damage [22]. Furthermore, these reactions are thought to occur preferentially on macromolecules associated with weakly chelated redox-active iron [77].

The specific cell death mechanisms triggered in tumor cells by high-dose ascorbate are not yet fully understood. Therefore, the role of ferroptosis in the ascorbate-induced death of cancer cells is unclear; presumably, depending on tumor entity and dosage, other forms of cell death also occur, such as autophagy and apoptosis [79]. Nevertheless, Wang et al. were able to demonstrate the induction of ferroptosis by high-dose ascorbate in anaplastic thyroid cancer cells [80]. Furthermore, it could be shown that ascorbate-induced accumulation of iron in combination with a simultaneous GSH reduction resulted in the enhancement of erastin-induced ferroptosis in pancreatic cancer cells [81].

## 3. Interplay between Ascorbate and Iron

Pharmacological ascorbate therapy affects the oxidation state of iron and increases free iron in the cytosol, which is a characteristic of various tumors [22,82,83]. Ascorbate mobilizes iron from ferritin by two separate processes: release of ferritin-bound iron by ascorbate alone or as labile iron citrate complex, which synergizes ascorbate-dependent iron mobilization and increases the maximum mobilization rate by about fivefold [84]. Under normal conditions there is a very low concentration of free iron, which is considered a source of continuous toxicity resulting in iron-ROS production. Since tumor cells are strongly dependent on iron intake for their growth and proliferation, the influx and efflux of iron through the cell membrane plays a crucial role in this process [85,86]. In contrast to iron import, most cells do not have an effective mechanism to export iron, resulting in an increase in LIP levels when the amount of iron exceeds the storage capacity thereby affecting cell survival [87]. Ferroptosis is an iron-dependent and lipid peroxidation-driven regulated cell death pathway [88,89]. In the field of redox biology, iron and other cationic metals such as copper also exacerbate oxidative stress in ferritin-containing tissue [84]. Therefore, in malignant cells, the electron-transferring properties enable labile iron to participate in the pro-oxidative reaction of ascorbate to form Asc•^−^, O_2_•^−^, H_2_O_2_, and OH• providing even more ferrous iron (Fe^3+^ + AscH^–^ → Fe^2+^ + H^+^ + Asc•^–^), boosting the Fenton reaction (Fe^2+^ + H_2_O_2_ → Fe^3+^ + OH•+ OH^–^) and enhancing pharmacological ascorbate-induced toxicity [22,31,82]. Hydrogen peroxide forms hydroxyl radicals under the catalytic action of Fe^2+^, which is the basis of free radical lipid peroxidation. Hydroxyl radicals and hydroperoxides are the two most widespread ROS that affect lipids, which may compromise the integrity of lysosomal membranes during oxidative stress [90,91,92]. Lipid peroxidation is a positive feedback chain reaction driven by ROS, including superoxide peroxides and free radicals, initiating the oxidation of polyunsaturated fatty acids [91]. As labile iron was shown to be closely related to ascorbate-induced toxicity for different cell types, the interaction between iron and ascorbate inside and outside cells seems to play an opposite role [82,93,94,95]. However, if the hydroxyl radical generated outside the cell by the Fenton reaction is already reacting extracellularly, it cannot reach its intracellular targets appropriately, which strongly reduces the anticancer efficacy of ascorbate [94,95]. Consequently, the time-shifted combination of iron with pharmacological ascorbate to increase the intracellular iron toxicity via enhancing the effect of pharmacological ascorbate is promising [82,93]. However, this requires the tumor cell to have sufficient capacity for the uptake of iron. Iron can generally be categorized by the chelate in which it is presented to the cell, either as transferrin (Tf)-bound iron (TBI) or non-Tf-bound iron (NTBI), depending on the following major players: transferrin receptors (TfRs), divalent metal transporter 1 (DMT1), and ferroportin 1 (FPN1).

In view of the explanation for ascorbate-induced cytotoxicity, the accumulation of ascorbate-related co-actors in the cell and the stimulation of the respective uptake mechanisms seem to be relevant aspects for further research on pharmacological ascorbate therapy. In general, the transport mechanisms of molecules into (and out of) the cell are: (i) diffusion (passive as O_2_ molecules or facilitated diffusion along a concentration gradient, through a protein channel such as aquaporins (AQPs), and through ion channels); (ii) primary or secondary active transport; and (iii) vesicle-mediated transport (e.g., endocytosis or exocytosis). The relevant shuttle mechanisms for effective pharmacological ascorbate treatment and ferroptosis induction in cancer are summarized in Table 1.

## 4. Aquaporins

AQPs are channel proteins from a larger family of major intrinsic membrane proteins and are widely distributed in human tissues with different localizations at cellular and subcellular levels [96,97]. Since some members of the AQP family facilitate the diffusion of H_2_O_2_, they are also named peroxiporins [154,155,156,157] and their function has been related to both volume regulation and ROS elimination [96]. The regulation of H_2_O_2_ permeation can also contribute to a resistant phenotype of tumors and peroxiporin activity could modify the cellular antioxidative defense system, thereby contributing to oxidative stress resistance [158]. AQP1, AQP3, AQP5, AQP8, AQP9, and AQP11 expression was reported in human tumors, and in some cases correlated with tumor grade, opening new diagnostic and therapeutic opportunities [56,101,159,160,161,162,163,164,165,166,167,168]. Therefore, peroxiporin expression was suggested to be an important determinant modulating cancer cell susceptibility to therapeutic H_2_O_2_ formation induced by pharmacological ascorbate [161,169]. H_2_O_2_ plasma membrane permeability was demonstrated to have significant variability across cell lines [170]. Although intracellular H_2_O_2_ concentration plays a key role in cellular susceptibility to adjuvant ascorbate therapy (Table 1), its overall contribution to the therapeutic effectivity of ascorbate is not clear [161].

## 5. Ascorbate Transporters

The entrance of vitamin C into the cell is determined by specific transporters. These belong to a family of nucleobase transporters and are highly conserved through evolution [110,171]. Human sodium-dependent vitamin C transporter (SVCT) 1 is encoded by the solute carrier family 23 member 1 (*SLC23A1)* gene that is mapped on chromosome 5 yielding a 598 amino acid polypeptide (Table 1). SVCT2, as a *SLC23A2* gene product from chromosome 20, is a 650 amino acid polypeptide [172]. Both can actively transport ascorbic acid against gradients by coupling its entry with sodium influx into the cell, thus maintaining the sodium gradient throughout the plasma membrane, which is provided by Na^+^/K^+^-ATPase [173]. Although plasma membrane SVCT2 is a Na^+^-dependent co-transporter, it also exhibits absolute dependence on Ca^2+^ or Mg^2+^. In contrast, intracellular SVCT2 is exposed to high ascorbic acid and low Na^+^, Ca^2+^, and Mg^2+^ and is a low-affinity transporter that lacks Na^+^ cooperativity [173]. SVCT1 is situated in the brush-border membrane of absorptive epithelial tissue of, e.g., kidney, intestine, liver, lung, and skin [109,110]. In humans, no loss of *SLC23A1* has been described to date. Different single nucleotide polymorphisms (SNPs) have been shown to weaken ascorbate transport and to reduce plasma levels with a trend toward a higher cancer risk for *SLC23A1* variants [111,174,175,176,177]. Heterogenic results were reported for gene expression control and there is also limited information regarding posttranslational modifications and the factors influencing cellular localization [60,178,179,180,181]. In cancer patients, studies evaluating SVTC1 tissue distribution between normal and malignant cells and also observations of SVTC1 expression under ascorbate or standard chemo-radiation regimens have not been conducted. SVCT2 is widely distributed and is therefore the predominant tissue transporter for vitamin C in most tissues and in blood cells [24,113]. A short isoform of SVCT2, naturally occurring in humans through alternative splicing, is unable to transport ascorbate and has the ability to partially inhibit SVCT1 [182,183]. Pathological circumstances associated with liver metabolic or oxidative stress may affect the expression of vitamin C transporters in different ways [178]. SVCT2 is expressed in Lewis lung tumors grown in ascorbate-dependent mice [184]. SVCT2 protein levels varied over time following a single high-dose ascorbate injection, but their association with tumor ascorbate levels was complex [184]. In human breast cancer cells, SVCT2 mRNA levels differed significantly between cell lines [185]. Cellular and subcellular localization of SVCT2 determines its transport activity and depends on different cell types, ascorbate concentration as well as intracellular Na^+^ and K^+^ concentrations [186,187,188,189]. Expression of SVCT2 in human neuroblastoma tissue was confirmed by immunofluorescence [190]. SVCT2 protein levels in breast cancer cells were predictive for ascorbate uptake and cellular sensitivity to ascorbate cytotoxicity [110]. This was confirmed by overexpression and gene knockdown in vitro [115]. Interestingly, SVCT2 expression was absent or weak in normal tissues but strongly detected in tumor samples obtained from breast cancer patients, suggesting that functional SVCT2 sensitizes breast cancer cells to autophagic damage by increasing the ascorbate concentration and intracellular ROS production. Therefore, the presence of SVCT2 in breast cancer may act as a predictor for the effectiveness of ascorbate treatment [115]. SCVT2 was overexpressed in the mitochondria of breast cancer cells, but only marginally presented on the plasma membrane [116]. Augmented expression of mitochondrial SVCT2 appears to be a common hallmark across all human cancers and might have implications for the survival capacity of cancer cells in pro-oxidant environments [116,191]. In addition, the analysis of numerous tissue microarrays contained in the Human Protein Atlas reveals the intracellular expression of SVCT2 in different cancer tissues [116]. Moreover, it was shown that resistance to cetuximab in human colon cancer patients with mutated Kirsten rat sarcoma viral oncogene homologue (KRAS) can be bypassed by ascorbate in an SVCT2-dependent manner. For the treatment of KRAS-mutated colon cancer, the SVCT2 expression may act as a potent marker for ascorbate co-treatment with cetuximab [28]. In addition, in low SVCT2-expressing cells, high-dose ascorbate (>1 mM) showed anti-cancer effects, but low-dose (<10 μM) (as defined by the authors for both incubation procedures) treatment induced cell proliferation in colorectal cancer cell lines so that insufficient uptake of ascorbate in low SVCT2-expressing cancer cell lines cannot generate sufficient ROS to kill the cancer cells [192]. Supplementation of Mg^2+^ enhanced the anticancer effect of ascorbate by inhibiting the hormetic response at a low dose, also providing a more pronounced anticancer response in cells with low SVCT2 expression compared to ascorbate treatment alone [193]. In hepatocellular carcinoma (HCC), the synergistic effect of ascorbate and sorafenib was shown in patients without elucidating the role of vitamin C transporters [194]. In cholangiocarcinoma cell lines, ascorbate worked synergistically with cisplatin [195]. Thereby, SVCT2 expression was inversely correlated with the half-maximal inhibitory concentration (IC_50_) values of ascorbate [196]. Furthermore, SVCT2 knockdown endowed cholangiocarcinoma cells with treatment resistance, and the SVCT2 expression level was suggested as a positive outcome predictor for ascorbate treatment in this tumor entity [195]. In the liver, a close relationship between B-cell lymphoma 2 (*BCL2*) and *SLC23A2* with several other genes was revealed to play an important role in the expression levels of these genes [197]. In line, a decreased ascorbate uptake mediated by SLC2A3 (alt. GLUT3) promotes leukemia progression and impedes ten-eleven translocation 2 (TET2) restoration [198]. High expression levels of SVCT2 were related to a good prognosis in patients with pancreatic adenocarcinoma [117]. Otherwise, only a limited association between ascorbate concentrations and its transporters was identified in renal cell carcinoma (RCC) cells and clinical samples [111]. Positron emission tomography (PET) imaging and tissue distribution analysis showed that cancer cells with high SVCT2 expression enhanced the accumulation of labeled ascorbate derivatives in mice after tumor formation. Correlations of *SLC23A2* gene polymorphisms related to ascorbate levels and disease risks depend on tumor entity and study population [175,199,200,201]. Two SNPs related to increased vitamin C plasma concentrations and several others were identified as posing a high risk of gastric cancer, follicular lymphoma, chronic lymphocytic leukemia, colorectal cancer (CRC), or head and neck cancer [175,176,177,199,201,202].

## 6. DHA Transporters

Ascorbate is taken up into cells via SVCTs. Since ascorbate easily oxidizes and transforms into DHA upon a pH change, DHA accumulates through facilitated diffusion via GLUTs [171,203]. The family of GLUTs consists of 14 members, which are encoded by the *SLC2* genes [204]. They are Na^+^-independent, ubiquitously distributed, and enable facilitated diffusion of glucose along its concentration gradient [119]. GLUT1, GLUT3, and GLUT4 are the specific glucose transporter isoforms that mediate DHA transport and subsequent accumulation of ascorbate [205,206]. Malignancies with activated hypoxia-inducible factor 1 (HIF-1) express high levels of glucose transporters such as GLUT1 [207]. Once in the cell, DHA is rapidly reduced to ascorbate, which is consumed by the cell [207]. KRAS or rapidly accelerated fibrosarcoma isoform B (BRAF) mutations that occur in colorectal cancer may also contribute to glucose uptake and GLUT1 overexpression (Table 1). Human colorectal cancer cells harboring KRAS or BRAF mutations were selectively killed in vitro when exposed to high levels of vitamin C [64]. This effect was due to increased uptake of DHA via GLUT1 [64]. Moreover, cancer cells were able to acquire vitamin C, even if they expressed an abnormal form of SVCT2, by using GLUTs and converting DHA into ascorbate [191], a phenomenon that is called the bystander effect [68]. Recently, it was shown that the uptake of DHA largely affects the redox metabolism of human erythrocytes [208], albeit these blood cells do not express SVCTs [24]. For non-small-cell lung cancer, it was demonstrated that ascorbate but not DHA is the cancer cell-selective toxic species and the latter was significantly less toxic [22]. Since tumors are regarded as complex heterogenic tissues with hypoxic areas [209], the Warburg effect is one of the hallmarks of cancer favoring the suppression of normal oxidative phosphorylation and the adaptation to hypoxia [210] via upregulating HIF-1 and GLUT expression [207]. Metabolic products such as lactic acid, originating in tumor cells [211,212,213], may promote the spontaneous oxidation of ascorbate to DHA due to pH lowering within the tumor microenvironment [32,33]. Both GLUT-mediated DHA uptake as well as enhanced ascorbate oxidation to DHA may be initiated in the tumor [214]. Currently, the role of GLUT-mediated DHA uptake in ascorbate-induced cytotoxicity appears to be only partially relevant, but this remains to be fully elucidated. Recently, GLUT3 was found to play a role distinct from that of GLUT1 in CRC, suggesting both prognostic value and therapeutic potential for GLUT3 expression [215]. Overexpression of GLUT3 was used for rapid DHA uptake and transformation into ascorbate followed by GLUT3 inhibition and attenuation of glucose uptake. This “suicide cycle” was considered to generate not only high levels of oxidative stress, which are harmful to CRC cells, but also an energetic crisis due to the blockade of glycolysis and reduced GLUT3-mediated glucose input. Therefore, CRC cells with high GLUT3 expression were found to be highly sensitive to treatment with vitamin C [215].

## 7. Transferrin Receptors

Extracellular Fe^3+^ is almost exclusively bound to Tf and reflects the essential insolubility of trivalent iron at physiological pH. Structurally, serum Tf is primed for Fe^3+^-binding upon release into the bloodstream [216]. Therefore, Tf prevents the hydrolysis and precipitation of the metal ion thus increasing its blood solubility and bioavailability. Serum Tf also inhibits the reduction of Fe^3+^ to Fe^2+^, which, if left uncontrolled, would lead to iron toxicity from the excessive production of ROS. For instance, there is some evidence that Tf can bind Fe^2+^ that enters the bloodstream, providing another Fe-based regulatory function of serum Tf. The presence of Fe^2+^ in serum is an indicator of a diseased state with loss of Fe-homeostasis. Tf may be able to rescue the unwanted Fe^2+^, rapidly converting it into Fe^3+^ via a ferroxidase-like mechanism [216]. TBI import starts by binding to a dimeric transmembrane glycoprotein as its receptor, which was also suggested as a specific ferroptosis marker [217]. There are two types of TfRs: TfR1, which is widely expressed and binds Tf with higher affinity, and the less-common TfR2, which is predominantly expressed in hepatocytes and erythroid precursors [137,138]. TfR1 is overexpressed on many different types of cancer cells, often at levels many times higher than in normal cells, which correlates with advanced tumor stage and poor prognosis [141]. TfR1 is a 90 kDa type II transmembrane glycoprotein consisting of 760 amino acids that is found as a dimer linked by disulfide bonds on the cell surface [139]. The TfR1 monomer is composed of a large extracellular C-terminal domain of 671 amino acids containing the Tf-binding site, a 28 amino acid transmembrane domain, and an intracellular N-terminal domain containing 61 amino acids [141]. Each subunit is capable of binding one free Fe^3+^ and Tf may thus have up to two atoms of iron attached. Diferric Tf or holo-Tf (two iron atoms bound to Tf) has the highest affinity (*K*_D1_ < 0.1 nM, *K*_D2_ = 3.8 nM, pH 7.4) compared to apo-Tf (Tf-lacking iron; *K*_D1_ = 49 nM, *K*_D2_ = 344 nM, pH 7.4) [137]. Since TfR1 is found as a dimer and can bind two Tf molecules, the receptor preferentially binds diferric Tf to the “bottom” of the TfR1 close to the cell membrane referred to as the “basal portion” and forms a ligand-receptor complex on the cell surface, which is constitutively internalized via clathrin-mediated endocytosis [139]. In general, TfR1 is expressed at low levels in most normal cells [144]. Numerous genes, such as transcription factors, growth factors, cytokines as well as HIF-1α, are involved in regulating the gene expression of TfR1 [141,144,218,219,220]. An association was shown between some pathophysiologic conditions and genetic alterations within the TfR [221,222,223,224,225]. However, there is limited evidence of an association between genetic variants of TfRs and cancer. Recently, the genetic susceptibility related to the hepcidin-regulating gene pathway, including TfR1 and TfR2, was shown to be associated with PDAC risk [226]. In many cancers, TfRs expression is significantly dysregulated, and iron uptake is abnormal [144]. The expression of TfR1 appears to be significantly higher in tumor tissues compared to adjacent non-cancerous tissues [142]. In this context, the expression of TfR1 and TfR2 negatively correlates with tumor differentiation. When TfR1 is significantly overexpressed, it correlates with tumor stage, and is associated with progression and poor prognosis, high risk of recurrence, and short patient survival [142,143,144]. In light of these analyses, TfR1 has been proposed as a prognostic marker for many tumors [227]. However, it should be considered that the role of TfR1/2 in tumor prognosis might be tumor-specific [228,229]. TfR2 is also frequently expressed in human cancer cell lines [230]. In vitro investigations of iron loading or iron deprivation provided evidence that TfR2 is modulated in cancer cell lines according to cellular iron levels. Iron loading caused two different mechanisms: in some cells a downregulation of total TfR2 and in other cell types a downregulation of membrane-bound TfR2, without affecting the levels of total cellular TfR2 [230]. In both conditions, iron deprivation caused the opposite effect compared to iron loading [230]. Mutations within TfR2 have functional consequences and cause hereditary hemochromatosis (HH) type 3 [231]. The prevalence of pathogenic TfR2 genotypes depends on ethnicity [146,232]. Moreover, some SNPs have been described to be associated with iron biomarkers [146].

## 8. Divalent Metal Transporter 1

DMT1 belongs to the *SLC11* gene family of metal-ion transporters that use the H^+^ electrochemical gradient (Table 1) [233]. DMT1 is expressed widely and accepts a broad range of transition metal ions as substrates, among which Fe^2+^ is transported with high affinity (K_0.5_ ≈ 2 μM). DMT1 accounts both for the intestinal absorption of free Fe^2+^ and for Tf-associated endosomal Fe^2+^ transport in erythroid precursors and many other cell types. In the intestine, DMT1 is up-regulated dramatically by dietary iron restriction and, despite high serum iron levels, it is not appropriately down-regulated in HH. DMT1 is highly expressed in various cancers such as colorectal cancer and ovarian cancer [129,130,131]. DMT1, TfR1, and ferritin were found to be highly expressed in ovarian cancer cell spheres and overexpression of DMT1 promoted the progression of ovarian tumors [129].

## 9. Ferroportin

The iron-efflux protein solute carrier family 40 member 1 (SLC40A1/FPN1) extrudes iron into the extracellular space and Fe^2+^ is re-oxidized to Fe^3+^ by ferroxidases outside the cell (e.g., ceruloplasmin (CP) or hephaestin (HEPH)). This exporter is localized to chromosome 2q and encodes a protein of 570 amino acids (Table 1) [128,234,235,236]. It has 12 putative transmembrane domains [237,238]. FPN1 is the major basolateral iron exporter in epithelial cells, highly expressed in the hepatic Kupffer cells, periportal hepatocytes, duodenal enterocytes, splenic red pulp macrophages, and the placental syncytiotrophoblasts [147]. The regulation of FPN1 expression is complex with important layers of control at transcriptional, posttranscriptional, posttranslational, and cell-lineage levels [147]. The primary method of FPN1 regulation is post-translationally via hepcidin [239]. Once hepcidin has bound to FPN1, it results in its ubiquitination, internalization, and degradation, playing a central role in the regulation of body iron levels [240]. Notably, FPN1 regulation varies among different cell types, allowing additional flexibility in controlling systemic iron flux under different conditions [128]. In the small intestine, FPN1 production is strongly regulated by the amount of iron and oxygen and increases the absorption of dietary iron during iron deficiency and anemia [147]. In contrast to hypoxia and iron deficiency, inflammation decreases the expression of FPN1 through effects on transcription [241]. In response to an increased iron load, the liver secretes the peptide hormone hepcidin, which binds to and induces internalization and degradation of the cellular iron transporter FPN1, thus controlling the amount of iron released from the cells into the blood [242]. FPN1 is the only cellular efflux channel for iron, resulting in a decrease in cellular iron output. Clinically detectable FPN1 mutations are very heterogeneous and can result in two phenotypically distinct diseases: HH type 4A and HH type 4B (FPN disease). HH type 4B is caused by gain-of-function mutations resulting in partial or complete hepcidin resistance [147]. Not all FPN1 mutations have been classified based on their phenotypic presentation or pathogenicity [147,243]. A systematic meta-analysis of FPN1 mutations found over 90 different variants among other disease-causing mutations [243,244] with ethnicity-dependent incidence [146,244]. Since cancer cells, with their high metabolic rates and rapid multiplication, have a particularly high requirement for iron, their FPN1 activity is downregulated, increasing the iron pool [151,152,153,245]. In contrast, stimulating FPN1 leads to reduced growth and proliferation of cancer cells due to cellular iron deprivation caused by increased activity of FPN1 [246,247,248]. Tumor-associated macrophages appear to have an iron-exporting phenotype which may provide iron to the cancer cells and further promote tumor growth [249].

## 10. Other Iron-Related Transport Systems

Under physiological circumstances, iron is incorporated into and transported by Tf in the blood, which safely sequesters the metal as Fe^2+^. However, under conditions of iron overload, the iron-binding capacity of plasma Tf can be exceeded, resulting in the appearance of NTBI which is a major contributor to the pathological iron loading of various tissues. [250,251,252]. This iron is then taken up by divalent ion transporters, which also transfer other metals, such as L-type and T-type calcium channels, the zinc-regulated transporter (Zrt)/iron-regulated transporter (Irt)-like proteins (ZIPs) 8 and 14, or transient receptor potential canonical channel 6 (TRPC6) [252,253], some of which are upregulated in cancer [254] or suppressed by mutated p53 [253]. ZIP8 (SLC39A8) was shown to be involved in the progression of neuroblastoma, metastasis [255], and sensitivity to chemotherapy [256]. ZIP14 is a member of the SLC39A zinc transporter family, which is involved in zinc uptake by cells and also transports NTBI and manganese [250]. Aberrant ZIP14 expression and altered zinc homeostasis were detected for PDAC [257]. ZIP14 transport activity can influence the intracellular concentrations of these ions through endocytotic trafficking [253]. TRPC6 protein is a nonselective cation channel permitting the uptake of essential elements such as iron and zinc and displays a large distribution profile detected in many organs and tissues [112]. However, it is necessary to clarify how the unique function of these transporters relates to the induction of ferroptosis and affects iron metabolism as well as the development and treatment options of cancer, especially in the context of ascorbate-mediated pharmacotherapy. In addition, there is evidence for an alternative mechanism of iron uptake. Epican (extracellular matrix receptor III, CD44), as a multifunctional cell surface adhesion receptor, is a regulator of the progression and metastasis of cancer cells [258] in the context of glycan-mediated iron endocytosis during EMT, in which iron operates as a metal catalyst to demethylate repressive histone marks that govern the expression of mesenchymal genes [259]. The role of pharmacological ascorbate treatment also has to be explored regarding the stem cell marker prominin 1 (PROM1, CD133) which downregulates TfR1-mediated endocytosis of diferric Tf [260], including CD91 and CD193. These are reported to be highly expressed in cancers and associated with a dismal prognosis [261,262,263]. Alternatively, PROM2, another member of the prominin family of pentaspan membrane glycoproteins, causes ferroptosis resistance by stimulating exosome-dependent iron export through the formation of ferritin-containing multivesicular bodies in epithelial and breast carcinoma cells [264]. Therefore, blocking the iron release pathway on cell membranes increases the susceptibility to ferroptosis. Furthermore, the inhibition of system Xc^−^, with decreased cystine uptake, glutathione depletion, and increased NAPDH oxidation, as well as the release of arachidonic acid mediators [87,92,265,266] as targeted cysteine deficiency can lead to ferroptosis [89]. As amino acids cannot diffuse directly into cells, they must be transported across the cell membrane with the help of specific transport proteins such as system Xc^−^, an amino acid antiporter responsible for the intracellular transport of extracellular cystine by exchanging intracellular glutamate. [267]. The amino acid antiporter SLC7A11 (xCT, system Xc^−^) is composed of two different core components: the light-chain subunit SLC7A11 (xCT) and the highly conserved heavy chain subunit SLC3A2 (4F2hc) [268]. Once inside the cells, cystine is reduced by system Xc^−^ to cysteine, an essential substrate for glutathione synthesis [89,267]. Under the conditions of extracellular oxidation, the exchange of cystine and glutamate is the most upstream event of ferroptosis. The inhibition of the SLC7A11 pathway may be the most critical mechanism for inducing ferroptosis [269,270].

## 11. Conclusions and Outlook

Pharmacologic ascorbate treatment was reported to have an anti-cancer potential over 40 years [12,13,14]. Some of the biological effects have been demonstrated only in models that are not directly transferable to humans. In the meantime, some pre-clinical investigations and a couple of small early phase clinical trials (phase I–II) have shown the feasibility, selective toxicity, tolerability, and potential efficacy of intravenous high-dose ascorbate therapy as is promising factors for the treatment of for different tumor entities [21]. However, the final evidence for the efficacy of high-dose ascorbate therapy in tumor patients is not yet conclusive. Although there are promising results from phase I and IIa studies, there is still a lack of proven results from larger and randomized patient cohorts such as phase III clinical trials. In addition to its antiproliferative effect on tumor cells, ascorbate also contributes to improved patient quality of life [21]. 

It is worth mentioning that there are also publications by Jara et al. [271] and Ramirez et al. [272] that postulate a proliferation-promoting effect of ascorbate on tumor cells through in vitro and in vivo results. Therefore, the authors advise against high-dose ascorbate therapy and point out the possible advantage of vitamin C deficiency in tumor patients. Thus, these results contradict the vast majority of in vitro and in vivo studies. However, these contradictory findings can be elucidated by the fact that Jara et al. only compare orally achievable plasma concentrations with vitamin C deficiency. Furthermore, the experiments of Ramirez et al. were partly carried out with DHA but not ascorbate, and the results of the highest investigated pharmacological ascorbate concentration of 500 µM showed no difference to physiological concentrations. In contrast to the observations of these two animal models, it is known from clinical findings that vitamin C deficiency is associated with a poorer prognosis in cancer patients [273].

**Figure 1:**
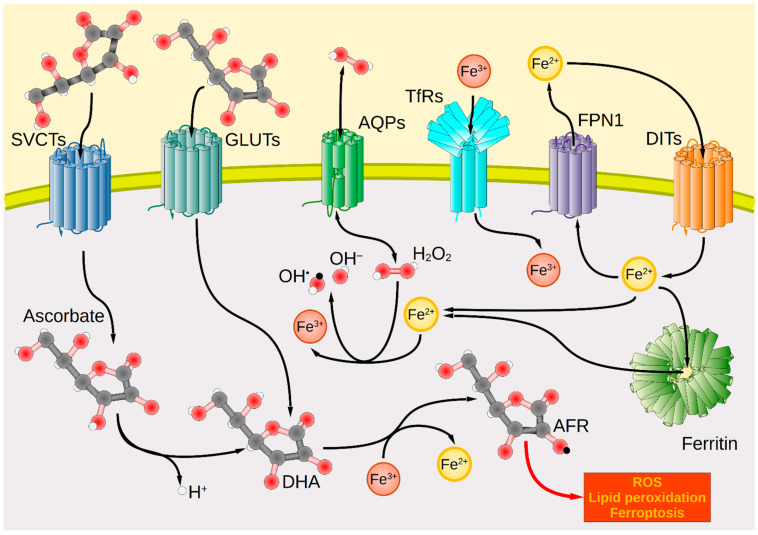
Transport systems for ascorbate, iron, and hydrogen peroxide across the plasma membrane and intracellular redox interactions in tumor cells. Cancer cells take up ascorbate via the transport proteins SVCT1 and SVCT2, whereas DHA uptake is mediated by GLUTs (GLUT1/3/4). Intracellularly, DHA is oxidized by Fe^3+^ to form the AFR, increasing cellular oxidative stress. Extracellularly formed H_2_O_2_ enters the cell interior through AQP membrane channels, where it forms hydroxyl radicals (OH•) under the catalytic action of Fe^2+^ as part of the Fenton reaction. These lead to the generation of further ROS, lipid peroxidation, and ultimately cell death. Iron import occurs by TfR via endocytosis for Tf-bound ferric iron (Fe^3+^) or in the case of unbound ferrous iron (Fe^2+^) by various DITs, e.g., DMT1. In contrast, iron export occurs through FPN1. Intracellularly, iron is present predominantly in the form of Fe^2+^ bound to ferritin. Red spheres symbolize oxygen atoms; black spheres carbon atoms; white spheres hydrogen atoms. Black arrows mark the direction of transport or reaction. AFR, ascorbate free radical; AQP, aquaporin; DHA, dehydroascorbic acid; DIT, divalent iron transporter; DMT1, divalent metal transporter 1; FPN1, ferroportin 1; GLUT, glucose transporter; ROS, reactive oxygen species; SVCT, sodium-dependent vitamin C transporter; Tf, transferrin; TfR, Tf receptor.

Nevertheless, there is currently no treatment option that allows the patient to be completely cured of the majority of tumors. Pharmacological ascorbate therapy could potentially provide room for optimization and synergism, especially considering that tumor patients often have scurvy-like low blood levels [5,274]. Regarding ROS generation, the interaction between iron and ascorbate plays an extraordinarily promising role. Data from cell models indicate that ascorbate forms AFR and H_2_O_2_, and reduces intracellular unbound iron that, in return, generates a greater amount of ROS [22]. As tumor cells exhibit increased iron metabolism there is an elevated iron uptake and diminished iron release contributing to the rise of LIPs [22,275,276]. For effective pharmacological ascorbate treatment and ferroptosis induction in cancer, vitamin C importers SVCT1 and 2, GLUT1 and 3, iron importers TfRs and DMT1, iron exporter FPN1, as well as AQPs as channel proteins, can be considered as relevant shuttle systems (Figure 1). 

**Figure 2:**
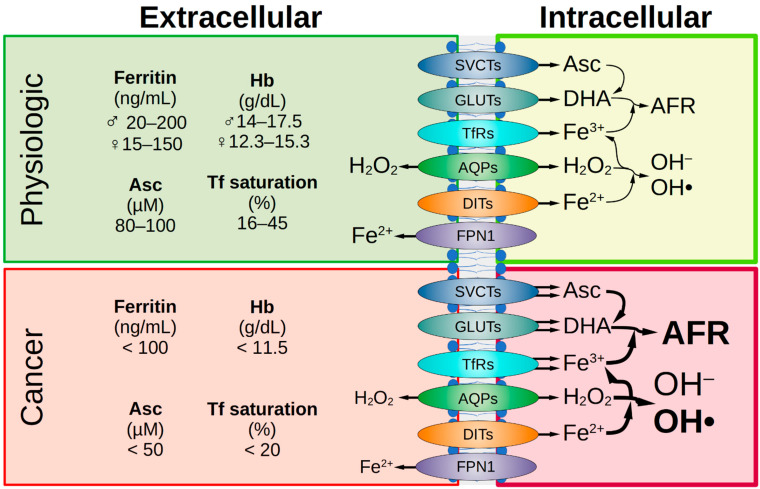
Comparison of healthy and malignant tissue. Under physiological conditions, iron status is represented by various extracellular parameters. The physiological ascorbate concentration is 80–100 µM. The transport of Asc, DHA, iron, and H_2_O_2_ across the cell membrane is facilitated by various transport proteins and receptors. Intracellularly, under physiological conditions, the amount of labile iron is low, therefore the formation of the AFR and hydroxyl radicals is low. In tumor patients, a decrease in iron parameters and ascorbate concentration in the extracellular space is often observed. If SVCT2 expression is high in the malignant cells, they are sensitive to ascorbate treatment and this is favorable for treatment success. Due to the frequently elevated expression of various GLUTs in tumor cells, there is increased uptake of DHA and enhanced formation of AFR. This is favored by an elevated pool of intracellular labile iron in cancer cells due to enhanced uptake via TfR as well as decreased iron efflux due to reduced FPN1 expression. This leads to supraphysiological H_2_O_2_ concentrations in cancer cells. Under pharmacologic ascorbate treatment (plasma levels up to 20 mM), H_2_O_2_ concentration is massively increased and causes cellular damage. AFR, ascorbate free radical; AQP, aquaporin; Asc, ascorbate; DHA, dehydroascorbic acid; DIT, divalent iron transporter; FPN1, ferroportin 1; GLUT, glucose transporter; Hb, hemoglobin; SVCT, sodium-dependent vitamin C transporter; Tf, transferrin; TfR, Tf receptor.

Ascorbate has a complex chemistry, enables the reduction of Fe^3+^, and facilitates iron uptake (Figure 2) [277].

In addition, ascorbate decreases the solubility of iron in the ferritin core, increases the exchangeable iron pool of ferritin, and thus additionally increases the net iron release rate of iron from ferritin [83]. Tumor cells have a limited capacity to regulate LIPs that react with pharmacological ascorbate or H_2_O_2_ and contribute to additional ROS formation [22,278]. Accumulation of unbound cellular iron and ROS elevation consequently results in excessive lipid peroxidation and additionally limits the antioxidant resources within the tumor cell, leading to gene and protein modifications as well as morphological changes, and results in ferroptotic cell death [275,279]. Noteworthy, the significance of iron in carcinogenesis has also been documented in experimental animal models [280,281] and abnormal iron levels in plasma and tissues are assumed to be directly associated with cancer [282]. On the other hand, anemia is a common complication in cancer patients, both at diagnosis and during treatment, with remarkable negative impacts on quality of life and overall prognosis. It is caused by multifactorial iron deficiency and may be present in almost half of all patients with solid tumors and hematologic malignancies [283]. The deregulated iron homeostasis is often found in malignant cancer phenotypes and makes cancer cells more sensitive to iron deficiency than normal cells, leading to increased iron affinity and supply as well as inhibition of iron release, thus contributing to iron accumulation [85]. Therefore, numerous approaches have been explored for the treatment of cancer targeting abnormalities in the intracellular iron metabolism [266]. The strategy, in contrast to iron depletion, is to supply cells with excess iron in combination with high levels of reactive iron and therefore cytotoxic levels of ROS [284]. For example, the metal-containing ferrocene derivatives are stable and exhibit favorable redox properties, and inhibit the proliferation of tumor cell lines [85]. Additionally, ferumoxytol is an iron oxide nanoparticle approved by the FDA for the treatment of clinical iron deficiency, and studies have shown that it can produce excessive amounts of free iron [85]. The resulting ROS can cause cell death, increase oxidative stress, and reduce tumor burden in mouse leukemia models and patients [85]. In addition to the above-mentioned iron-related anti-tumor strategies, another therapeutic approach is to directly target membranous iron transporters [266,284]. In this context, the availability of uptake systems on the tumor cell surface and the knowledge about their functionality is important to achieve sufficient ascorbate and iron levels to induce antiproliferative effects. An aberrant expression and a divergent genetic background have already been described for the individual ascorbate or iron transporters and for certain tumor entities [118]. Currently, there is still a lack of knowledge regarding whether a coordinated interaction among the individual uptake mechanisms to the detriment of the tumor cells is prevalent or can be therapeutically initiated. At least for co- and pre-incubation with iron and high-dose ascorbate in vitro, it is already known that in vitro co-incubation with iron decreases the prodrug activity of ascorbate [82,93,94,95]. The role of peroxiporins, which could shuttle released hydrogen peroxide into the cell, is also unclear. Nevertheless, the establishment of extracellular hydrogen peroxide levels to achieve target intracellular hydrogen peroxide levels is important to optimize the dose and duration of intravenous ascorbate, especially since low intracellular hydrogen peroxide levels could even promote tumor growth whereas high intracellular concentrations are considered antitumoral [285]. In the blood, the achieved hydrogen peroxide levels seem to be significantly lower (<50 nM) than in the extracellular space because of degradation by red blood cell or plasma proteins [35]. In general, hydrogen peroxide formation is influenced by many extracellular and intracellular factors [34,286]. Regarding the increased demand for iron in cancer cells, the consideration of non-Tf-bound iron uptake systems as alternative routes may be a promising target for investigation (Figure 2). In addition, the tumor ecosystem, including substances and degradation products as well as oxygen supply and temperature, is crucial for the optimal functionality of each transporter [266,277,287]. The generated ascorbate concentration gradient also supports the passive ascorbate transport, which depends on the plasma membrane potential and pH and determines the steady-state intracellular ascorbate concentration [288].

Moreover, it is important to also evaluate the impact of the used chemotherapeutic agents and irradiation, in vitro and in animal models, on the expression and functionality of the membrane uptake systems related to the high-dose ascorbate-induced cytotoxicity. The multidrug resistance extrusion systems on the cell surface which are inducible due to chemotherapeutic treatment as a protection against cytotoxic agents and cellular survival strategy should also not be neglected [289,290]. Besides the ROS induction and capability of disrupting intracellular iron metabolism, the anticancer activity of pharmacological ascorbate includes its modulation of SVCT2 activity by adding Mg^2+^, acting on tumor stem cells, and modulating epigenetics due to a hormetic response [25,193,291]. In summary, there is an urgent need for a nuanced understanding of the interplay between ascorbate, hydrogen peroxide transporters, and iron shuttle systems for sufficient ferroptosis induction, and it is necessary to identify tumors sensitive to ascorbate therapy to further substantiate the success of this application (Figure 2).

## Figures and Tables

**Table 1:** Major proteins involved in the transport of H_2_O_2_, vitamin C, and iron across the cell membrane and their expression in normal and tumor tissues.

Protein Name	Substrate	Tissue Expression	Tumor Tissue Expression	Functionally Relevant Genetic Polymorphisms	Consequences of Genetic Variations	Association with Other Parameters	References
AQP1/3/5/8/9/11	H_2_O_2_	widespread, e.g., lung, kidney, pancreas	various, e.g., pancreatic cancer breast, ovarian, prostate cancer, CRC, HCC, glioblastoma	AQP9: SNP rs1516400	associated with chemotherapy response in lung cancer patients	AQP1 expression is associated with increased DFS in CRC patients and AQP3 expression with OS in gastric cancer patients	[96,97,98,99,100,101,102,103,104,105,106,107,108]
				AQP11: SNP rs2276415	associated with kidney disease in type 2 diabetic patients		
SVCT1	vitamin C	epithelial tissue of kidney, liver, intestine, lung, skin	RCC	SNPs rs33972313 and rs11950646	decreased vitamin C plasma concentration	-	[109,110,111,112]
				SNPs rs659647 and rs11950646	higher risk of follicular lymphoma		
SVCT2	vitamin C	widespread, e.g., CNS	various, e.g., breast cancer, melanoma, CRC, pancreatic cancer	multiple SNPs	increased vitamin C plasma concentration, increased risk of gastric cancer, follicular lymphoma, leukemia, colorectal adenoma	-	[24,113,114,115,116,117,118]
GLUT1/3/4	DHA	ubiquitous, e.g., brain, placenta, prostate	widespread, e.g., CRC, HCC, prostate cancer, lymphoma, glioblastoma, lung cancer	GLUT1: SNP rs710218	increased risk of CRC, associated with susceptibility to develop clear-cell renal carcinoma	increased expression of GLUT1 is associated with unfavorable OS and poorer DFS	[64,119,120,121,122,123,124,125,126,127]
DMT1	Fe^2+^	ubiquitous, strong expression e.g., in proximal duodenum, brain, kidney, placenta	various, e.g., CRC, ovarian cancer, prostate cancer, esophageal adenocarcinoma	SNP 1254T>C	associated with Parkinson’s disease	high expression is associated with longer DFS in HCC patients	[128,129,130,131,132,133,134,135,136]
				SNP IVS4+44C/A	associated with increased blood levels of iron, lead, and cadmium		
TfR1/2	Fe^3+^ (Tf- bound)	widespread, e.g., liver, intestine, activated immune cells	various, e.g., HCC, breast cancer, ovarian cancer, pancreatic cancer, lung cancer	multiple SNPs	associated with iron biomarkers	TfR1 expression correlates with tumor stage and is associated with a high risk of recurrence and short patient survival	[137,138,139,140,141,142,143,144,145,146]
				SNP rs9846149	reduced risk for gastric cancer		
FPN	Fe^2+^	liver, duodenum, placenta, bone marrow, breast, brain	reduced activity in most tumor entities, e.g., cholangiocarcinoma, breast cancer, pancreatic cancer, prostate cancer	gain of function mutations	hepcidin resistance, HH type 4B	decreased FPN expression is associated with reduced survival in breast cancer patients	[147,148,149,150,151,152,153]

AQP: aquaporin; CNS: central nervous system; CRC: colorectal cancer; DFS: disease-free survival; DMT1: divalent metal transporter 1; FPN: ferroportin; GLUT: glucose transporter; HCC: hepatocellular carcinoma; HH: hereditary hemochromatosis; OS: overall survival; RCC: renal cell carcinoma; SNP: single nucleotide polymorphism; SVCT: sodium-dependent vitamin C transporter; Tf: transferrin; TfR: Tf receptor.

## Abbreviations

AFR, ascorbate free radical; AOC, advanced ovarian cancer; AQP, aquaporin; Asc, ascorbate; BCL2, B-cell lymphoma 2; BRAF, rapidly accelerated fibrosarcoma isoform B; CP, ceruloplasmin; CRC, colorectal cancer; DHA, dehydroascorbic acid; DMT1, divalent metal transporter 1; EMT, epithelial-mesenchymal transition; FPN1, ferroportin 1; GBM, glioblastoma; GLUT, glucose transporter; GSH, glutathione; GSSG, GSH disulfide; HEPH, hephaestin; HH, hereditary hemochromatosis; HIF-1, hypoxia-inducible factor 1; IC50, half-maximal inhibitory concentration; Irt, iron-regulated transporter; KRAS, Kirsten rat sarcoma viral oncogene homologue; LIP, labile iron pool; NAD+, nicotinamide adenine dinucleotide; NADP+, nicotinamide adenine dinucleotide phosphate; NOX, NADPH oxidase; NTBI, non-Tf-bound iron; PARP, polyADP-ribose polymerase; PET, positron emission tomography; PDAC, pancreatic ductal adenocarcinoma; PROM, prominin; ROS, reactive oxygen species; SCLC, small cell lung cancer; SLC23A1, solute carrier family 23 member 1; SNP, single nucleotide polymorphism; SOD, superoxide dismutase; SVCT, sodium-dependent vitamin C transporter; TBI, Tf-bound iron; TET2, ten-eleven translocation 2; Tf, transferrin; TfR, Tf receptor; TNBC, triple-negative breast cancer; TRPC6, transient receptor potential canonical channel 6; ZIP, Zrt/Irt-like protein; Zrt, zinc-regulated transporter.
